# Ice Cores as a Source for Antimicrobials: From Bioprospecting to Biodesign

**DOI:** 10.34133/bdr.0024

**Published:** 2023-11-02

**Authors:** Ying-Chiang Jeffrey Lee, Bahar Javdan

**Affiliations:** ^1^ Department of Molecular Biology, Princeton University, Princeton, NJ 08544, USA.; ^2^ Rutgers Robert Wood Johnson Medical School, New Brunswick, NJ 08901, USA.

## Abstract

The golden age has passed for antibiotic discovery, and while some antibiotics are currently in various phases of clinical trials in the United States, many pharmaceutical companies have abandoned antibiotic research. With the need for antibiotics, we should expand our horizon for therapeutic mining and can look toward understudied sources such as ice cores. Ice cores contain microorganisms and genetic material that have been frozen in time for thousands of years. The antibiotics used by these organisms are encoded in their genomes, which can be unlocked, identified, and characterized with modern advances in molecular biology, genetic sequencing, various computational approaches, and established natural product discovery pipelines. While synthetic biology can be used in natural product discovery approaches, synthetic biology and bioengineering efforts can also be leveraged in the selection and biodesign of increased compound yields, potency, and stability. Here, we provide the perspective that ice cores can be a source of novel antibiotic compounds and that the tools of synthetic biology can be used to design better antimicrobials.

## Introduction

Antimicrobial resistance (AMR) is a major global health threat and is estimated to contribute to 10 million human deaths by 2050 [1]. Despite this, many large pharmaceutical companies have eliminated or reduced their antibiotic research footprint. AMR genes are not new, and have evolved over millions of years, and continue to evolve due to selective pressures, making finding novel antibiotic compounds a pressing task. One potential source of new antibiotics is ice cores. Ice serves as a biological repository, providing a record of microbial evolution, harboring biological specimens dating back many millennia [2,3]. Microorganisms that have adapted to the extreme environment and are able to survive serve as a unique source of biomolecules, which have allowed for the development of unique physiology and metabolic pathways [4–6]. These features could provide expanded set of options that antimicrobials target and reveal compounds that could be adapted for current use. For example, different cell wall architectures could necessitate stronger antimicrobial peptides that penetrate bacterial cells. Cold-adapted microbes have already been shown to produce antimicrobial compounds, making them a novel resource to find molecules to fight AMR strains [7,8]. In addition, bacteria not necessarily adapted to the cold, but found frozen in ice, also offer a lens to microbe–microbe interactions that can be examined for therapeutic use. In a 13,000-year-old ice core sample, AMR and evidence of antimicrobial activity have been observed [9].

According to the Baas Becking hypothesis, microbes are everywhere but nature selects [10]. We have been traditionally been searching for antimicrobial compounds from soil and marine environments but to a lesser degree in cold environments where nature has selected for different microorganisms. In this perspective, we outline a research workflow where ice cores can be mined for their antibiotic compounds and synthetic biology approaches can be used to further optimize aspects of production as well as compound design.

## Bioprospecting of Ice Cores

The potential antimicrobial-mediated ecological interactions locked within ice cores can be revealed through examination of genetic records and attempts to culture the microbes themselves. Antagonistic interactions between organisms in the form of predator–prey relationships can put selective pressure on prey and give rise to species with defensive molecules that may potentially have antimicrobial activity. Antimicrobials can also be derived from symbiotic relationships where one organism secretes antimicrobial compounds that protect a host from pathogens. The ecological as well as nonlethal, alternative, effects of sub-inhibitory levels of antimicrobials have been discussed in more detail by others [11,12]. Here, we propose harvesting these compounds for therapeutic uses.

In the ice core metagenomic analysis (ICEMAN) pipeline, samples are examined from a sequencing, metagenomic approach as well as from a laboratory culturing approach. The sequence-based protocol, termed antibiotic discovery and analysis of metagenomes (ADAM), can be executed in a complementary fashion to the culturing-based environmental examination (EVE) protocol, thus maximizing the potential for recovering antibiotic compounds.

ADAM relies on the assembly of DNA reads that can construct an accurate metagenomic representation. This is then subject to the examination by multiple computational programs that detect signatures of antibiotic genes such as MetaBGC [13] MetaBGC, originally developed for drug prospecting in the microbiome, detects biosynthetic gene clusters (BGCs) encoding microbial metabolites at the single read level and is especially useful when microbes are poorly represented in metagenomic data or have low abundance—situations that are likely applicable to bioprospecting within ice cores. Other programs relying on machine learning algorithms to identify antimicrobial peptides can also be used. In addition to identifying dedicated antibiotic compounds, cryptically encoded peptides within larger proteins should be considered. Recent research has demonstrated that such cryptic peptides can be isolated and tested against pathogenic organisms successfully [14–16]. ADAM would also include bioactivity-guided product discovery from metagenomic libraries, which has been shown to identify metabolically important proteins [17]. Taken together, ADAM would provide a list of possible antibiotic compounds at the small-molecule and peptide level that can be candidates for experimental validation.

While the ADAM approach relies on sequenced genetic information, EVE seeks to directly sample ice cores for extant microbial life that could be metabolically active provided an optimal growing environment. Organisms are known to enter cryptobiosis due to adverse environmental conditions. Recent research has shown that nematodes and viruses from permafrost can be revived and cultured [18,19]. Through rigorous culturing methods that include supplying relevant growth and stimulatory factors, revived microbes could then be screened for antimicrobial properties using traditional antimicrobial assays. In some cases, coculturing may be needed. Compounds of interest can then be isolated using chemical extraction techniques and characterized using mass spectrometry and nuclear magnetic resonance. Revived organisms could also be used to construct genomes that would be appropriate for further analysis using tools in the ADAM approach.

## Synthetic Biology Approaches

While synthetic biology approaches can be used to help identify ice core-derived antimicrobial compounds, synthetic biology can also be used to help optimize antibiotics against AMR through design and selection [20]. The list generated from the ADAM and EVE represents compounds of interest that can be further engineered by applying biodesign and drug development principles. Many innovative methods have recently demonstrated utility for high-throughput screens of peptide-based compounds such as using phage display or outer membrane protein tethers [21,22]. These approaches can help select for low toxicity, high efficacy, and low immunogenicity.

Synthetic biology approaches such as heterologous gene expression, pathway and metabolic engineering, and spatial programming can be used to not only produce and identify ice core-derived antimicrobial compounds but also up-regulate production in downstream purification processes [23]. For example, potential antimicrobial products from genetic fragments, BGCs, or predicted genes could be produced using inducible promoters on plasmids with variable copy number that are inserted into heterologous hosts. In addition, cell-free systems could be used in certain cases where products are toxic. Biosynthesis of other antimicrobial compounds could also rely on the presence, and optimization, of various enzymes. Such synthetic biology approaches have been used in the production and engineering of natural products such as terpenoids, alkaloids, polyketides, as well as nonribosomal and ribosomal peptides [24–28].

## Conclusion

Microorganisms within ice cores are a possible repository for novel antimicrobial compounds that could be used in our ongoing struggle with AMR. Here, we outline the ICEMAN pipeline that incorporates both sequence-based and culture-based approaches to identify antibiotic compounds of interest in an orthogonal manner, maximizing the potential for antibiotic discovery from ice cores. Synthetic biology approaches can be applied for initial compound production as well as optimization steps.

A point of consideration in the context of reviving ancient microbes is that of biosafety and biosecurity. Organisms and viruses originating from samples thousands of years old should be treated with care and following best practices in molecular biology to ensure the safety and security of not only the researchers handling the samples but also the public and current biosphere. Environmental contamination by an unsuspecting pathogen could be a threat to human, animal, and plant health. ICEMAN research should be conducted with proper oversight and handling protocols. ICEMAN taps into the ecological and genetic potential within ice cores, and a focused effort to mine and characterize antibiotic compounds within these samples could contribute to the health and wellness of society.
